# DAGs and GRaSP Causal Inference Algorithms Combined and Applied to the Calculation of Insulin Bolus in Patients with Type 1 Diabetes

**DOI:** 10.3390/e28050506

**Published:** 2026-05-01

**Authors:** Rocío Contreras-Jiménez, Juan Carlos Olivares-Rojas, Adriana del Carmen Téllez-Anguiano, Jesús Eduardo Alcaráz-Chávez, José Antonio Gutiérrez-Gnecchi, Enrique Reyes-Archundia

**Affiliations:** División de Estudios de Posgrado e Investigación, Tecnológico Nacional de México/I. T. de Morelia, Morelia 58120, Mexico; rocio.cj@morelia.tecnm.mx (R.C.-J.); adriana.ta@morelia.tecnm.mx (A.d.C.T.-A.); eduardo.ac@morelia.tecnm.mx (J.E.A.-C.); jose.gg3@morelia.tecnm.mx (J.A.G.-G.); enrique.ra@morelia.tecnm.mx (E.R.-A.)

**Keywords:** artificial intelligence, causal inference, DAGs, GRaSP, health technology, insulin bolus calculation, type 1 diabetes

## Abstract

Type 1 diabetes mellitus (T1DM) is a chronic, non-preventable, and incurable disease that requires lifelong insulin administration. The principal challenge is calculating the prandial insulin bolus to avoid hypoglycemia and hyperglycemia. Traditional bolus calculators are based on limited number of variables, but there are many variables that define the complex interactions among glucose levels, like carbohydrate intake, physical activity, mood, and contextual factors. While recent artificial intelligence (AI) approaches have shown promise in glucose prediction, most remain correlational and offer limited interpretability for clinical decision support. This study evaluates a causal inference-based framework for insulin bolus calculation using Directed Acyclic Graphs (DAGs) and the Greedy Relaxation of the Sparsest Permutation (GRaSP). Historical data from individuals with T1DM were analyzed, incorporating domain knowledge constraints to guide structure learning. A bootstrap-based stability analysis was conducted to evaluate the robustness of inferred relationships. Results show that integrating prior medical knowledge reduces graph complexity and improves interpretability. However, bootstrap stability reflects robustness of the learning procedure rather than causal validity. The findings suggest that the proposed framework is useful for generating plausible causal hypotheses, but not for confirming causal relationships. Further validation using conditional independence testing, equivalence class analysis, and temporal causal methods is required. However, the proposed framework focuses on generating plausible causal hypotheses rather than establishing causal validity, which requires further refutation-based validation.

## 1. Introduction

T1DM is a chronic autoimmune disease characterized by the destruction of pancreatic beta cells, leading to absolute insulin deficiency and requiring lifelong exogenous insulin administration. Maintaining postprandial glucose levels within a safe range is essential to prevent acute complications such as hypoglycemia and diabetic ketoacidosis, as well as long-term microvascular and macrovascular damage [1,2] and other complications like blindness from diabetic retinopathy, kidney failure, heart attacks, brain stroke, and foot ulcers or amputations. It also causes neuropathy, sexual problems, and an increased risk of infections. All the above cause mental diseases and economic problems for the family and for the public health systems. A central challenge in T1DM management is calculating the prandial insulin bolus, which must balance multiple interacting physiological and behavioral factors. This implies high costs for patients and their families, the public health system, and the patient’s economically active life [3].

### Background

Conventional bolus calculators are primarily based on fixed carbohydrate-to-insulin ratios and correction factors. They use traditional formulas and are often updated infrequently, or only when a correction is needed. Carbohydrate Counting (CC) is a systematic method for calculating insulin bolus size; in addition to improving metabolic control, CC has been shown to improve quality of life [4]. Often, these approaches struggle to adapt to interindividual variability and to the complex, nonlinear relationships present in real-world data. In recent years, AI and Machine Learning (ML) techniques have been proposed to improve glucose forecasting and insulin decision support.

Over the past decade, several studies have incorporated AI techniques to automate and personalize bolus calculation. Among them, Noaro, Capon et al. used quadratic LASSO regression (LASSO\(_Q\)) to improve dose estimation, reducing the frequency of hypoglycemia in simulations [5]; Zhu, Taiyu et al. implemented deep reinforcement learning (DRL) to optimize prandial dosing, increasing time in range (TIR) in adults and adolescents [6]; Kalita et al. [7] developed InsNET, a deep neural network capable of predicting basal and bolus doses with high accuracy, Yoo, Pradana et al. proposed an algorithm combining offline reinforcement learning (RL) with an online fine-tuning technique [8]. There are algorithms whose functions are embedded in loops close to insulin pumps, such as automated insulin delivery systems that integrate continuous glucose monitoring with algorithm-based insulin dosing [9]. Although these systems have shown promising results, they share a fundamental limitation: they are black boxes. These correlational models prioritize prediction accuracy over interpretability, understanding the underlying physiological mechanisms, and identifying the causes of postprandial hyperglycemia, all of which depend on the patient’s context and the insulin bolus administered. However, most of these methods are fundamentally correlational, offering limited interpretability and little guarantee of causal validity, which is important in clinical settings where patient safety is paramount. In contrast, causal inference provides tools to model underlying mechanisms and generate hypotheses about cause–effect relationships.

This study contributes by: integrating domain knowledge into GRaSP-based causal discovery for T1DM, evaluating structural stability under bootstrap resampling, and providing a framework for hypothesis generation in insulin dosing.

## 2. Theoretical Framework

Causal inference provides a framework of principles for moving beyond prediction and understanding the underlying mechanisms governing glycemic dynamics and the variables involved. In this context, causal algorithms (CAs) seek to infer causal relationships (CRs) from observational patient data, thereby encoding valid causal relationships between variables [10]. Among these methods, the GRaSP algorithm has established itself as a scalable and theoretically sound approach, particularly well-suited to high-dimensional and potentially dense graphs, which are common in biomedical and digital health data [11]. Considering the above, the main aim of this work is to explore and evaluate the application of DAGs and GRaSP. DAGs are graphical representations used to encode causal assumptions among variables. They are not algorithms per se, but structural models that can be learned or specified. In contrast, GRaSP is a causal discovery algorithm that searches over the space of possible DAGs using a score-based approach. In this project, DAGs and GRaSP were used separately and in combination to calculate insulin boluses in T1DM. By integrating medical-domain constraints and stability analysis, this study assesses whether CD can improve the structural coherence and interpretability of data-driven models, thereby supporting personalized insulin dosing analyses while acknowledging the inherent limitations of observational data.

### 2.1. DAGs and Causal Discovery

Directed Acyclic Graphs (DAGs) are graphical representations used to encode causal assumptions among variables. They are not algorithms, but structural models that describe causal relationships. “*DAG is a type of graphic which nodes are linked by unidirectional connections that aren’t part of any cycle. DAGs are used to illustrate the dependencies and the causal relations*” [12].

Causal discovery (CD) algorithms aim to infer such structures from data. Among them, GRaSP is a score-based algorithm that searches over permutations of variables to identify sparse graph structures. DAGs provide a mathematical structure to identify causal relationships, enabling intervention and counterfactual reasoning. Unlike predictive Machine Learning Models (MLM), causal inference can determine whether a variable, in this case, bolus insulin, truly affects an outcome, postprandial glucose, which can contribute to better control of glucose in DBT1 patients [13,14].

### 2.2. GRaSP Algorithm

GRaSP is a family of CD algorithms that explore the space of variable permutations to construct parsimonious DAGs and use local operations, such as tuck, to iterate toward more parsimonious solutions. GRaSP introduces a hierarchy of relaxations: GRaSP 0, 1, 2. This offers point-consistency guarantees under progressively weaker assumptions than faithfulness, and benchmarks demonstrate good accuracy and scalability compared to other AI methods [11].

GRaSP (Greedy Relaxation of the Sparsest Permutation) is a causal discovery algorithm that explores permutations of variables and applies local operations to identify Directed Acyclic Graphs (DAGs) that optimize a scoring criterion.

GRaSP explores the permutation space of variables; given a permutation, it constructs “sparse” DAGs consistent with that ordering and uses local operations—tucks and swaps—to traverse the permutation space, searching for permutations that yield more parsimonious DAGs. This combines the advantages of ordering-based and score-based methods. GRaSP comes with point-consistency guarantees under the assumptions established by the authors; furthermore, in simulations, the algorithm demonstrated competitive performance against contemporary methods in terms of accuracy and scalability for more than 100 variables. GRaSP is designed to be computationally efficient compared to exact graph searches; practical implementations and utilities have also been published (official repository and documentation in benchmarking tools and CD libraries) [11,15].

In this study, GRaSP was implemented using the SEM-BIC score, which assumes linear Gaussian relationships among variables. Therefore, preprocessing steps were applied to approximate Gaussianity.

### 2.3. Comparison of GRaSP with Other CD Approaches

Against methods based on independence tests (e.g., Peter Clark algorithm [16], Fast Causal Inference [17]): GRaSP is score- or ordering-based, so it is generally more robust to Causal Inference test errors in high-dimensional or complex dependency scenarios. However, this depends on the choice of score and the conditional family model [11].

Compared with other score-based methods like Fast Greedy Equivalence Search (FGES) [18], Greedy Sparsest Permutation (GSP) [19], or Non-combinatorial Optimization via Trace Exponential and Augmented lagRangian for Structure learning (NOTEARS) [20,21] algorithms. In benchmarks, the more relaxed version of GRaSP was competitive or superior in several scenarios, especially when the graph was dense; however, relative performance can vary depending on sample size, noise, and the presence of deterministic or nonlinear correlations. Additional evaluations show that methods such as Greedy Fast Causal Inference (GFCI), FGES, or GRaSP can produce dense graphs in specific temporal or autocorrelated scenarios [11].

### 2.4. Healthcare Applications—Where Is CD Useful?

The CD field is gaining greater attention due to its potential to apply CD methods in the biomedical sciences, but it remains underleveraged. The high interpretability provided by causality enables more reliable decision-making and high-quality intervention procedures for precision medicine [22].

### 2.5. DAGs in Healthcare

Recently, the DAGs have been used in healthcare, for example, to evaluate the associations between metal mixtures and cardiometabolic outcomes [23] and to identify confounders in the association between coronary heart disease and pesticide exposure among greenhouse vegetable farmers [24]. Since 2017, there have been many investigations in healthcare using DAGs because of their flexibility [20] and their ability to explicitly represent causal relationships in complex biomedical systems, as in this case, where glucose regulation involves multiple interacting variables. DAGs allow these relationships to be modeled as a structured causal system. DAGs allow us to demonstrate various properties of conditioning on a common effect. By incorporating sufficient causes into a graph, it is possible to detect conditional causes [25]. DAGs allow us to: identify real cause-and-effect relationships, distinguish confounders, mediators, and collisions, and avoid incorrect inferences. This is crucial because insulin is not only correlated with glucose, but it also causally modifies it. DAGs allow the identification of the minimum set of variables necessary to estimate causal effects. One of the main problems in traditional or purely predictive models is the inclusion of unnecessary variables, the exclusion of critical variables, and the introduction of bias. DAGs allow the application of the d-separation criterion to identify the minimum set of causal fit.

**Figure 1:**
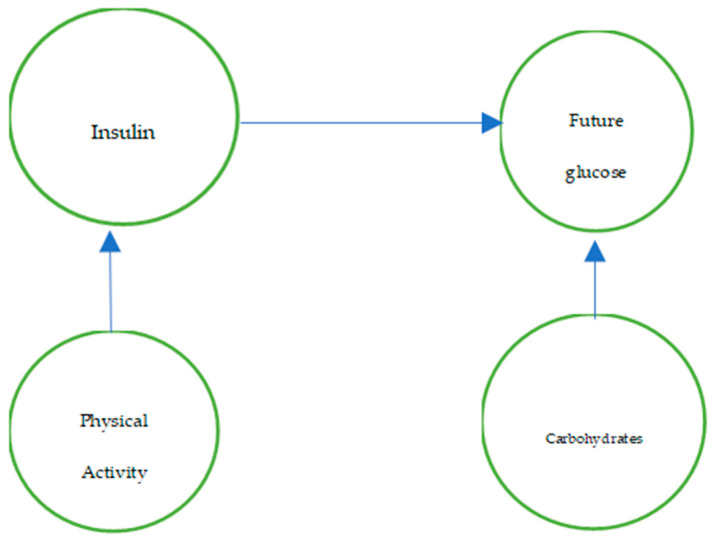
Example of variable dependencies.

This is crucial for bolus calculation because it allows identification of truly necessary variables and those that patients can record in their glucose diaries, generating a historical dataset of diary registers, such as shown in Figure 1:

A diagnosis-assisted glucose analysis allows us to determine which variables should be considered to accurately estimate the effect of insulin on postprandial glucose.

### 2.6. GRaSP Algorithm Use

Although GRaSP was proposed in the general context of CD, its applicability to healthcare is promising in several domains: Biomedical/omics network inference (genes, metabolites, proteins): The need to identify causal relationships in high-dimensional data makes permutation-based search attractive, and variants of GRaSP/GRASP are used in biological network inference. Furthermore, GRaSP may be part of the set of algorithms evaluated for constructing causal/functional graphs in omics [15,26].

Electronic Health Records (HER) and clinical observational studies: GRaSP is a candidate for uncovering causal dependencies between treatments, biomarkers, and clinical outcomes in large electronic databases (due to its scalability compared to dense graphs); its use has been reported or included in CD pipelines in recent studies applying algorithms to complex data, studies from 2024 to 2025 that integrate GRaSP into comparative analyses or analysis workflows [27].

Analysis of biomedical time series and physiological signals: Although GRaSP was designed for contemporaneous variables, some extensions and comparisons include it to estimate contemporaneous relationships in temporal CD/contemporary edges studies but there are caveats that specialized temporal methods can outperform GRaSP if the variables or scoring are not tailored [28].

Recent preprints and works (2024–2025) already employ GRaSP as part of their CD pipelines for applied problems (e.g., papers from 2025 that use it in interaction analysis contexts and in works that combine CD and counterfactual reasoning). These examples demonstrate that the method is being applied in practical settings, including domains closely related to medicine and digital health [27].

### 2.7. Practical Recommendations for Applying Causal Inference to Health Problems

- Preprocessing: imputation and treatment of categorical and continuous variables, standardization and transformation of non-Gaussian variables, if the score assumes normality. Control and labeling of time and lagged variables.
- Selection of GRaSP variants. Test the variants of the algorithm GRaSP (0, 1, 2) and compare them with FGES and GSP; the more relaxed variant is usually more robust if you suspect faithfulness violations [11].
- Evaluation and Validation: Use simulations with possible structures to assess sensitivity/specificity. Contrast the findings with expert medical knowledge and the interventions in this case; we expected to conduct a trial with real patients and to validate the findings with endocrinologists.
- Software and reproducibility: Implementations and materials from the paper will be available in the repository and code when the investigation ends, and GRaSP integrates into benchmark frameworks, facilitating reproducible testing [15].

## 3. Materials and Methods

This study follows an observational, retrospective design using real-world data collected from individuals diagnosed with T1DM. The dataset includes time-stamped records of preprandial glucose measurements, carbohydrate intake, insulin bolus doses, basal insulin rates, physical activity indicators (steps), heart rate, estimated caloric expenditure, and postprandial glucose outcomes. All data were de-identified before analysis.

### 3.1. Study Design and Data Source

The dataset used in this study is HUPA-UCM from a public repository. The data was acquired from 25 people with T1DM. CGM data were collected with FreeStyle Libre 2 CGMs, and Fitbit Ionic smartwatches were used to collect glucose levels, carbohydrate intake, insulin doses, and physiological variables for at least 14 days [29].

### 3.2. Ethical Considerations

The study uses secondary, anonymized observational data and does not involve direct intervention with human participants. According to local institutional guidelines, formal ethical committee approval was not required. Nevertheless, the study adheres to the principles of the Declaration of Helsinki and to best practices for research involving human-derived health data.

### 3.3. Variables and Preprocessing

The primary variables considered in the analysis include preprandial glucose level, carbohydrate intake, insulin bolus dose, basal insulin rate, physical activity (steps), heart rate, caloric expenditure, and postprandial glucose level. This last variable was calculated in the program two hours after a carbohydrate intake.

Data preprocessing involved handling missing values via listwise deletion for CD, as recommended for score-based methods, and standardizing continuous variables and normalizing the dataset. Non-Gaussian variables were examined and transformed when necessary to satisfy the modeling assumptions of the selected scoring function. No imputation was applied to outcome variables to avoid introducing artificial dependencies.

### 3.4. CD Framework

A causal model is an abstract quantitative representation of real-world dynamics. Therefore, a causal model attempts to describe the causal and other relationships between a set of variables [30].

The reason for choosing causal inference in insulin bolus calculation is not that it has not been used for this purpose before, but rather that the association and correlation of variables that has been applied so far in most existing Machine Learning algorithms is that causality allows us to make interventions and counterfactuals, which could help us detect causal inference in observational data without putting the health of a real patient at risk. Causal structure learning was performed using DAGs representation and the GRaSP algorithm. Domain knowledge was incorporated as constraints to restrict implausible relationships.

### 3.5. Methodology

**Figure 2:**
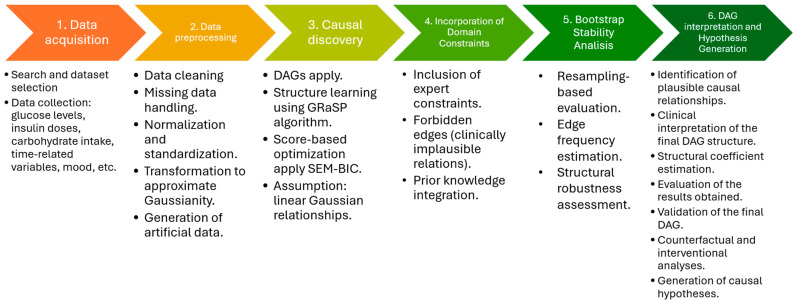
Methodology of the investigation.

The methodology applied in this investigation consisted of the steps shown in Figure 2, which combines the data science steps with additional steps.

### 3.6. Model Assumptions

GRaSP was implemented using the SEM-BIC score, which assumes linear Gaussian relationships among variables. Therefore, all variables were transformed to approximate Gaussianity prior to model estimation.

For the implementation details, the GRaSP algorithm was implemented using available tools from the Tetrad project [31]. The SEM-BIC score was used as the optimization criterion.

In the stability analysis, a bootstrap procedure was applied to assess structural stability. It is important to note that bootstrap evaluates the robustness of the learned structure under resampling, but does not provide evidence of causal validity. Bootstrap analysis was used to estimate the frequency of edge selection, which reflects the stability of the learned structure under resampling. It is important to note that, as was said, this measure does not imply causal validity.

Given the longitudinal nature of the data, the row-wise bootstrap may overestimate stability. Alternative approaches, such as patient-level bootstrap or temporal block bootstrap, are proposed for future work, along with a separate evaluation of edge presence and edge orientation stability.

To clarify the role and contribution of each methodological component, Table 1 summarizes their inputs, outputs, and specific functions within the proposed framework. This distinction helps distinguish among structure learning, causal effect estimation, and stability analysis.

### 3.7. Use of Generative Artificial Intelligence

Generative artificial intelligence tools were used exclusively to assist with language editing, clarity, and formatting of the manuscript text. No GenAI systems were used for data generation, data analysis, model development, or result interpretation. Therefore, the scientific content, analyses, and conclusions remain entirely attributable to the authors.

## 4. Results

### 4.1. The First Steps

**Figure 3:**
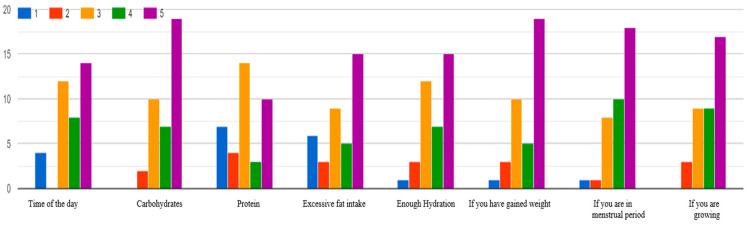
Example of the results of the survey applied to medical personnel of the IMSS Michoacán.

**Figure 4:**
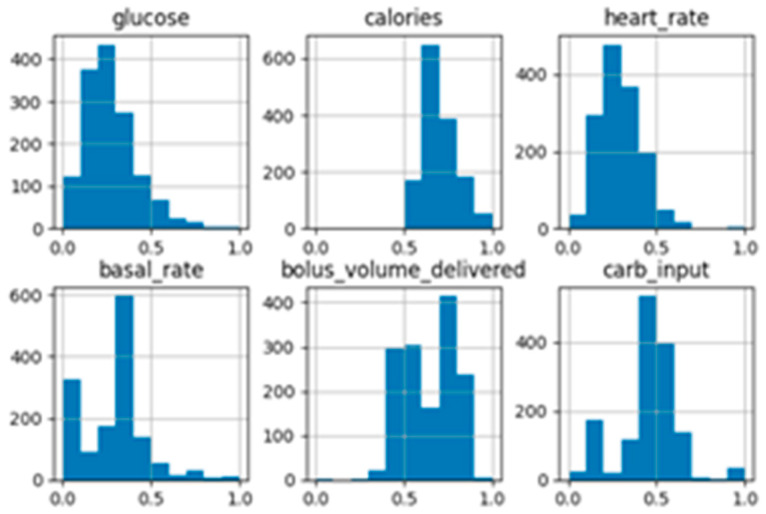
Example of distribution graphics after two transformations of the data.

1. In the initial steps, the problem to solve was defined: calculate the amount of insulin that a patient with T1DM should administer, based on the identification of patterns in historical data through the use of artificial intelligence models, allowing the patient to have better control and avoid as much as possible the occurrence of hyperglycemia (glucose levels above 180 mg/dL) or hypoglycemia (glucose levels below 70 mg/dL).
2. Once the problem was defined, a review of the state of the art on bolus insulin calculation using ML methods and AI was conducted. This research found that the majority of investigations have used traditional methods, relying on correlations rather than analyzing the causes, variables, or weights of those variables in postprandial glucose.
3. To identify variables that influence postprandial glucose and can be monitored by patients or caregivers, a survey was applied to 40 medical personnel at the *Instituto Mexicano del Seguro Social* (IMSS) in Michoacán, and the following principal variables were obtained: the correct doses of insulin, weight, body mass index (BMI), if the patient is a teenager, the hour of the day when the glucose is taken, amount of carbohydrates that were consumed, dehydration, sedentary lifestyle, quantity and intensity of the exercise that the patient performs, among other variables that were evaluated with lower values but are also important. Figure 3 shows an example of the results of this survey:

4. Begin the search for a dataset that has those variables or as many as possible. The HUPA-UCM dataset [29] was then selected. This dataset contains the data shown in Table 2:

5. The dataset was cleaned: incomplete, empty, or null records were removed. The postprandial glucose variable was added. This variable was calculated in the same dataset by searching for the glucose measurement two hours after the preprandial glucose measurement, carbohydrate consumption, and the bolus_volume_delivered.
6. Data normalization: data normalization and standardization were performed by establishing numerical values for categorical variables. The time variable was converted into two variables: date and time, and both were converted into seconds. The values of all variables were converted to the range [0, 1]. Then, the records with 0 in the variable bolus_volume_delivered were deleted, which represented the bolus of insulin administered to the patient’s principal foods, and the records with 0 in the variable carbohydrates were also deleted, because they did not represent a record of a principal food. At this point, the dataset was clean, normalized, and standardized. Then the distribution graphs were generated to understand the distribution of the data and the asymmetry of the probability distribution in each variable. This analysis showed that some variables require transformation.
7. Data transformation: the data was analyzed, and variables that do not follow a normal distribution were found, like: calories, steps, carb_input, heart_rate, glucose, and glucose_postprandial. These variables were transformed using the log and Yeo–Johnson transformation methods; later, the graphical results were analyzed, and a second transformation was applied using the sqrt and log methods; one example of the resulting graphics is shown in Figure 4.
8. Using AANN, artificial data was generated, with the original cleaning and normalized data, with the dataset of the first transformation, and with the second transformation, resulting in three datasets based on the original data, and three datasets with artificial data. However, AANN-generated artificial data were explored for robustness, but not used for causal validation or inference, and are not central to the conclusions of this study because the results obtained with the synthetic datasets were less accurate than those obtained with the normalized, standardized, and transformed datasets. Therefore, it was identified that the data generated did not respect the existing causal relationships. Consequently, an algorithm will be developed to generate synthetic data while preserving the identified causal relationships.

9. Algorithms selected: research on the CD algorithms, their uses in healthcare, advantages, and disadvantages were conducted, and six algorithms were selected. In this article, a DAG, which is a graphical representation used to encode causal assumptions among variables, and GRaSP algorithm will be presented, with the results of their application in the insulin bolus calculation.

**Figure 5:**
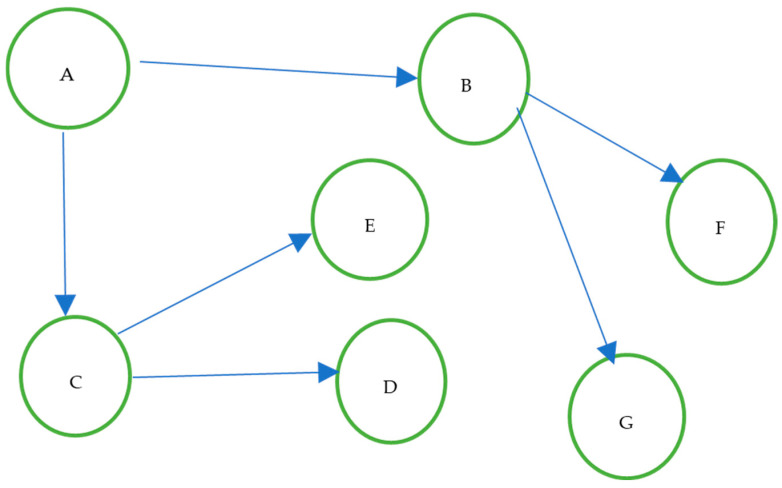
Example of DAG, the letters are only for variable examples.

“A DAG is a type of graph in which nodes are linked by unidirectional connections that do not form any cycle. DAGs are used to illustrate dependencies and causal relationships” [12]. The DAGs are composed of nodes representing variables that influence our final result, postprandial glucose (glucose two hours after consuming food), and arrows or edges that represent a unidirectional relationship. They are connections between variables or entities, which, when directed, are traversed in only one direction and represent that one node has a causal relationship with another, as can be seen in Figure 5.

GRaSP is a score-based CD method that searches over permutations of variables and applies local reordering operations (including the *tuck* operator) to identify parsimonious DAGs. This work employs the GRaSP relaxation level, which allows weaker assumptions than strict faithfulness and has demonstrated favorable performance in dense, high-dimensional settings [11].

**Figure 6:**
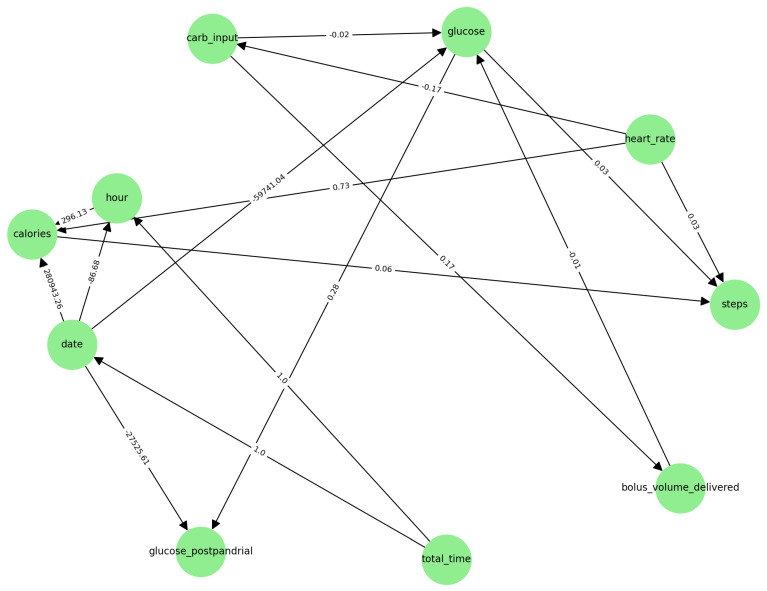
DAG based on adjustment knowledge.

10. Application for DAGs: In this phase, three DAGs were obtained: the first based on prior knowledge, the second on data, and the third on adjusted prior knowledge. The final DAG is shown in Figure 6.

11. This DAG was used as the basis for the GRaSP algorithm because, in the third model, results indicate that, considering multiple physiological and contextual factors, the insulin bolus has a significant negative effect on postprandial glucose. That is, it consistently reduces blood glucose after eating. Compared with the first model based on prior knowledge, where the estimated effect was stronger (around −1.30), the effect here is more moderate. This suggests that the third model reduces bias by adjusting for more relevant variables. The observed change in effect magnitude may reflect improved adjustment for confounding variables. However, it may also depend on the assumed graph structure and should therefore be interpreted with caution.

Given a candidate variable ordering, the DAGs consistent with that ordering were constructed by minimizing a sparsity-based score. Iterative local moves were then applied to improve the score. The Bayesian Information Criterion (BIC) was used as the scoring function, consistent with prior applications of GRaSP in biomedical contexts.

After cleaning and normalization, three versions of the dataset were generated: the original, logarithmically transformed, and Yeo–Johnson transformed. The GRaSP algorithm was implemented with the SEM-BIC score and the Fisher Z test of independence. To improve causal validity, knowledge constraints were applied to prohibit illogical relationships between variables, taking as input the DAG generated in the tenth step of the methodology. The generated structures were visualized using Graphviz [32] and validated using bootstrapping methods and causal effect estimates from DoWhy and EconML.

12. Obtaining causal parameters: this model identifies the backdoor and general adjustment variables in GAD, which are mathematical expressions:

Backdoor:

*d/d[bolus_volume_delivered] (E[glucose_postpandrial|date, calories, heart_rate, carb_input, steps, total_time, glucose, basal_rate, hour]).*

This means that the model will estimate: the causal effect of bolus_volume_delivered on postprandial glucose, adjusted for the listed variables. This method is based on the backdoor criterion, which is the most common and intuitive in causal models. It controls all variables that could confuse the relationship between insulin and postprandial glucose.

The formula represents: “the derivative of the expected value of postprandial glucose given bolus and adjustment variables.” Adjustment variables (conditioning): date, calories, heart rate, carb input, steps, total time, glucose, basal rate, and hour. These are the variables for which the model is statistically adjusted to block potential confounding pathways between bolus volume delivered and postprandial glucose. This allows for estimating a causal effect rather than a simple correlation.

Key assumption: Unconfoundedness.

*If U→{bolus_volume_delivered} and U→postprandial_glucose then P(postprandial_glucose|bolus_volume_delivered, ..., U) = P(postprandial_glucose|bolus_volume_delivered, ..., ).*

This means that: if there are unobserved variables U that affect both insulin and postprandial glucose, it is assumed that adjusting for these variables eliminates this bias. This is a strong assumption. It requires that you have measured all relevant variables that simultaneously affect insulin and postprandial glucose (confounders). If any important variables were left out (e.g., stress, sleep, alcohol), the estimate could be biased. In our case, we do not have these variables; they are variables we know exist but are unknown to us; however, we know they affect postprandial glucose.

General Adjustment variable:

Under the assumption that the adjustment set satisfies the general adjustment criterion, this expression can be interpreted as an estimate of the causal effect.

*d/d[bolus_volume_delivered] (E[glucose_postpandrial|steps,date,hour,heart_rate,glucose, total-time, basal_rate, carb_input, calories])*

Estimand assumption 1, Unconfoundedness:

*If U→{bolus_volume_delivered} and U→glucose_postpandrial then P(glucose_postpandrial|bolus_volume_delivered,steps,date,hour,heart_rate,glucose,total_time,basal_rate,carb_input,calories,U) = P(glucose_postpandrial|bolus_volume_delivered,steps,date,hour,heart_rate,glucose,total_time,basal_rate,carb_input,calories)*

A refutation test in this GAD with the method “random_common_cause” was performed, and the results were:

*Refute: Add a random common cause*

*Estimated effect: 0.054320*

*New effect: 0.054363*

*p value: 0.94*

A high *p*-value (in this case, 0.94) in a refutation test means that the test failed to refute the original causal estimate. In other words, adding a random unobserved common cause did not significantly change the estimated causal effect. This is a positive outcome, as it suggests that the causal estimate for this GAD model is robust and less likely to be confounded by unobserved variables that behave like the random common cause introduced. This increases confidence in the causal effect estimate under the assumptions and is consistent with the causal hypothesis.

### 4.2. Results and Incorporation of Domain Knowledge

To ensure clinical validity, domain constraints derived from established medical knowledge were imposed. Specifically, constraints were applied to avoid unlikely causal directions, for example, postprandial glucose as the cause of carbohydrate intake. These constraints were implemented as forbidden edges during graph construction, based on the previously generated DAG. This hybrid approach seeks to balance data-driven discovery with an expert-based structure.

### 4.3. Stability Analysis

The GRaSP algorithm was applied to the previously obtained DAG. To assess the robustness of the inferred causal structures, a bootstrap resampling procedure was used. The GRaSP algorithm was applied to multiple resampled datasets, including normalized, standardized, and transformed data, and edge selection frequencies were calculated. Only edges exceeding a predefined stability threshold were retained for interpretation. This approach reduces sensitivity to sampling variability and facilitates the generation of more stable causal hypotheses.

### 4.4. Software and Code Availability

All analyses were performed using open-source software. The DAG algorithm was implemented using DoWhy 0.14 and EconML libraries in Python 3.12.13 via Google Colab [33]. The GRaSP algorithm was implemented using the public GRaSP repository and integrated into the causal learning framework. Custom Python scripts were developed for preprocessing, constraint specification, and bootstrap analysis. The GRaSP implementation used in this study is available in the Tetrad project (version 7.4.0), which provides tools for causal discovery using score-based and constraint-based methods [34].

All code necessary to reproduce the results, including preprocessing routines, model configuration files, and analysis scripts, will be published in a dedicated repository upon completion of this research.

### 4.5. Standard GRaSP Structural Learning

**Figure 7:**
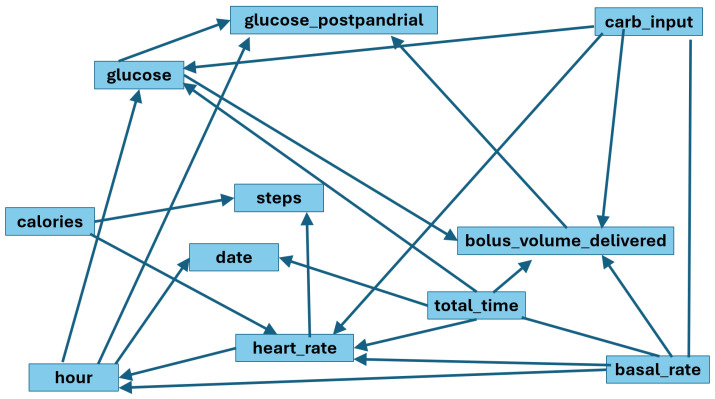
DAG output by standard GRaSP.

The standard GRaSP algorithm was applied without restrictions based on medical knowledge. The resulting DAG contained 22 directed relationships between the studied variables, as shown in Figure 7, some of which we might consider spurious at first glance, such as heart_rate or calories implying steps.

The learned structure identified direct associations between:

- Preprandial glucose and postprandial glucose.
- Insulin bolus (bolus_volume_delivered) and postprandial glucose.
- Carbohydrate intake and insulin bolus (bolus_volume_delivered).
- Exercise-related (steps) variables and glucose dynamics.

However, the unconstrained model presented edges that are not clinically plausible, suggesting potential violations of the Markov assumption or misspecification due to non-Gaussian distributions in some variables.

These findings indicate that purely data-driven CD may generate structurally dense graphs but with limited clinical interpretability.

### 4.6. Knowledge-Constrained GRaSP

To improve structural consistency, medical constraints derived from domain expertise were incorporated into the GRaSP search process. The constrained model produced a DAG with 12 directed relationships.

Compared with the unconstrained model, the knowledge-guided graph showed:

- Reduced structural density.
- Elimination of clinically implausible edges.
- More interpretable directional structure consistent with domain knowledge between insulin, carbohydrate intake, and postprandial glucose.

The reduced number of edges suggests that prior medical constraints improved parsimony and interpretability while preserving clinically meaningful dependencies.

**Figure 8:**
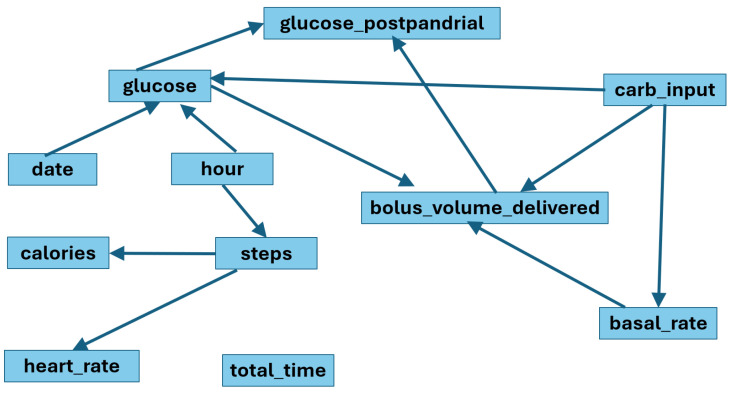
DAG output by a Bootstrap Run of 1000 folds with adjustment knowledge.

Based on this analysis, the DAG constructed from adjustment knowledge was used to impose restrictions on the DAG generated by the GRaSP algorithm. Figure 8 shows the DAG output by a Bootstrap Run of 1000-folds with adjustment knowledge (12 total relationships):

The estimated effect should be interpreted as a causal effect under the assumed DAG and unconfoundedness assumptions.

### 4.7. Bootstrap Stability Analysis

A bootstrap procedure of 1000 samples was performed to evaluate structural stability. The DAG with bootstrap constraints showed greater consistency in the central relationships involving:

- Insulin bolus → Postprandial glucose.
- Carbohydrate intake → Postprandial glucose.
- Preprandial glucose → Postprandial glucose.

Nevertheless, some variability was observed in secondary edges, indicating sensitivity to sample size and distributional assumptions.

These results suggest that incorporating knowledge from the medical domain improves structural robustness but does not completely eliminate instability due to limited sample size or possible incorrect model specification.

The unconstrained GRaSP model produced dense graphs with some implausible edges. Incorporating domain knowledge reduced the number of edges and improved interpretability.

Bootstrap analysis showed consistent presence of key relationships such as:

- Insulin bolus → Postprandial glucose
- Carbohydrate intake → Postprandial glucose

However, these results reflect structural stability rather than confirmed causal effects.

Table 3 shows the resume of the bootstrap stability analysis.

Bootstrap results indicate that core relationships, such as glucose → postprandial glucose, glucose → insulin bolus, and steps → heart rate, remain strong dependencies across resamples.

However, edge orientation showed variability, consistent with the limitations of observational causal discovery.

### 4.8. GRaSP Structural Learning (Core Results)

Table 4 shows the comparative analysis between the models: Model 1: Unconstrained GRaSP, Model 2: Constrained GRaSP, and Model 3: Bootstrap GRaSP. This table summarizes the structural differences between the unconstrained, knowledge-constrained, and bootstrap-based GRaSP models.

The incorporation of domain knowledge reduced the number of edges and eliminated clinically implausible relationships, improving interpretability without removing key dependencies.

## 5. Discussion

The results demonstrate that integrating medical domain knowledge into GRaSP improves the parsimony, structural consistency, and clinical interpretability of learned structures and reduces spurious relationships. This hybrid approach enabled more clinically consistent interpretations compared with purely data-driven models. However, several limitations must be acknowledged. While the unconstrained model captured multiple dependencies, it also introduced edges that could reflect statistical associations rather than valid causal mechanisms.

The learned DAG represents one member of a Markov equivalence class. Therefore, edge orientations are not uniquely identifiable from observational data.

The bootstrap analysis underscores the importance of assessing stability in high-dimensional biomedical settings. Violations of normality (Gaussianity) and potential deviations from causal sufficiency could explain residual structural variability, which represents the portion of variance in an endogenous (dependent) variable that is not explained by the explanatory (causal) variables included in the model. In causal terms, it is the part of the variation in the “effect” that is due to unmeasured factors, external influences, or unmodeled randomness [35]. Bootstrap stability does not imply causal validity; it only reflects the robustness of the algorithm.

The estimated effects under the assumed causal structure depend on the assumed graph structure and should be interpreted cautiously.

Future methodological refinement should focus on:

1. Parameter tuning of GRaSP scoring functions.
2. Validation of the final DAG using interventional or counterfactual analysis.
3. Testing conditional independence implied by the DAG (d-separation).
4. Estimation of structural coefficients for causal effect quantification, Completed Partially Directed Acyclic Graphs (CPDAGs), which are a graphic representation that is used in CI to describe a DAG’s equivalence class. They represent the structures of conditional dependencies that can be learned from observational data, and from Partial Ancestral Graphs (PAGs), which are graphical representations used in causal inference to represent the equivalence classes of Maximal Ancestral Graphs (MAGs), specifically when latent confounders or selection biases may be present. Unlike Directed Acyclic Graphs (DAGs), which assume causal sufficiency (no hidden common causes), PAGs are designed to handle observational data in which unmeasured variables might influence relationships [36]
5. Use of FCI/Greedy Fast Causal Inference (GFCI) to account for latent confounding.
6. Comparative evaluation against predictive models such as eXtreme Gradient Boosting (XGBoost) [37].
7. Temporal causal modeling.

### Causal Refutation Strategy and Validation Limitations

While the proposed framework improves structural interpretability and stability, it does not establish causal validity. To address this limitation, we outline a refutation-oriented validation strategy for future work.

The conditional independences implied by the final DAG can be derived using d-separation criteria. Those independences should be empirically tested in a validation dataset. Systematic violations would indicate that the proposed structure is inconsistent with the observed data. In this project, this was observed with the synthetic data; therefore, the results from the synthetic datasets were not used.

Invariance testing can assess whether the conditional relationships between variables remain stable across environments. In this context, environments may include different patients, time windows, or preprocessing configurations. If the parents of a target variable are truly causal, the corresponding conditional distribution should remain approximately invariant.

Temporal validation is required. The outcome variable (postprandial glucose) is defined with a time delay relative to its predictors. However, the current model is largely contemporaneous. Future work should incorporate lagged variables and temporal causal discovery methods to better capture dynamic relationships.

Equivalence class analysis should be incorporated. The learned DAG represents one member of a Markov equivalence class. Therefore, estimating CPDAGs or Partial Ancestral Graphs (PAGs), as well as using algorithms such as FCI, RFCI, or GFCI, would enable assessment of edge-orientation uncertainty and potential latent confounding.

Finally, causal effect estimation should be supported by robustness checks, including consistency across multiple valid adjustment sets, use of negative controls, and sensitivity analysis for unmeasured confounding.

These strategies are necessary to move from a plausible, stable structure to a more rigorously validated causal model.

## 6. Conclusions

GRaSP constitutes a modern, theoretically grounded alternative within CD methodologies. In insulin bolus estimation, incorporating knowledge-based constraints improves graph parsimony, structural interpretability, and bootstrap stability.

However, causal conclusions remain contingent upon model assumptions, data quality, and validation through intervention-based analyses. GRaSP should therefore be integrated within a broader causal inference framework that includes stability analysis, methodological triangulation, and clinical validation. In this case, GAD, based on prior medical knowledge, can be an alternative to avoid spurious causal relations between variables. The approach improves interpretability and structural consistency when incorporating domain knowledge.

However, the results should be interpreted as generating plausible causal hypotheses rather than confirming causal relationships.

## 7. Future Work

Although the proposed framework provides a structured approach for modeling causal relationships in postprandial glucose regulation, several relevant aspects remain open for further development.

First, work will be done on developing an algorithm that generates synthetic data that respects the causal relationships in the original data to achieve greater accuracy. A theoretically justified definition of the optimal parameterization for the GRaSP algorithm is still pending. This includes a systematic sensitivity analysis to determine how hyperparameter selection influences the learned causal structure and the robustness of the resulting Directed Acyclic Graph (DAG).

Second, the final causal DAG requires rigorous validation. Future work will include both internal validation (e.g., stability analysis under resampling and perturbation strategies) and external validation using independent patient datasets to assess structural consistency and generalizability.

Third, it is necessary to formally estimate and interpret the model’s structural coefficients in the GRaSP algorithm. This involves specifying the weights associated with each causal relationship and quantifying the corresponding noise (error) terms in GRaSP. Such parameter identification will allow the transition from a purely structural representation to a fully specified Structural Causal Model (SCM). This is because, in the application of GAD with the DoWhy libraries, structural coefficients were obtained, but they are not yet used by the GRaSP algorithm; they must be applied to the structure obtained and validated.

Furthermore, an important pending objective is to derive the complete causal structure and the associated system of linear equations that represent the joint probability distribution of all variables, conditioned on the target variable (postprandial glucose) and the treatment variable (postprandial insulin bolus). In the context of data from patients with diabetes, this formulation aims to explicitly model how the postprandial insulin bolus, together with relevant covariates—such as carbohydrate intake, pre-meal glucose levels, and other metabolic indicators—causally influences postprandial glucose dynamics.

Given the problem of generating synthetic data that does not preserve the causal relation between the original variables, an analysis explicitly allowing for nonlinear relationships could be considered, since the current analysis assumes linearity and Gaussianity, and the possibility that the data does not satisfy those assumptions is raised. If the relevant methodology is not currently available or effective, it may be in the future. By way of relaxing assumptions, it is also worth noting that GRaSP has been shown to be effective in the linear non-Gaussian case, and that recent versions of Tetrad include the Grow Shrink Trees optimization, which allows GRaSP to scale to well over 100 variables [38].

In the final stage of this research, the validated causal structure will be used to conduct counterfactual and interventional analyses. Specifically, the structural equations will enable the computation of do-interventions and counterfactual scenarios to evaluate hypothetical insulin administration strategies and their projected impact on postprandial glucose levels.

Finally, future research will focus on developing a causal predictive model for recommending postprandial insulin boluses. Unlike purely associative predictive models, this approach will incorporate the learned causal structure to generate treatment recommendations grounded in interventional reasoning, potentially improving personalization and clinical interpretability.

## Figures and Tables

**Table 1:** Summary of methodological components and their roles within the proposed framework.

Component	Role	Input	Output	Purpose	Validation Scope
AANN	Synthetic data generation	Observational data	Artificial dataset	Explore patterns and support robustness tests	Exploratory, non-causal
Dowhy (DAG)	Causal effect estimation	DAG and Observational data	Estimated causal effect	Quantify the effect under assumptions	Assumption-dependent
Prior DAG	Knowledge encoding	Clinical expertise and literature	Structural constraints	Reduce implausible relationships	Conceptual with domain-based
GRaSP	Structure learning	Observational data with constraints	DAG	Discover dependency structure	Statistical with score-based
Bootstrap (GRaSP-based)	Stability analysis	Observational data	Edge selection frequencies	Assess the robustness of the learned structure	Structural stability

Source: Own elaboration.

**Table 2:** Variables from the HUPA-UCM dataset from the Universidad Complutense de Madrid.

Field	Type
Time	Object
glucose	Float64
calories	Float64
heart_rate	Float64
steps	Float64
basal_rate	Float64
Bolus_volume_delivered	Float64
Carb_input	Float64

Font: Own elaboration with the information from [29].

**Table 3:** Results of Bootstrap stability Analysis.

Edge	Orientation Stability	Selection Frequency	Clinical Interpretation
Glucose → Bolus_volume_delivered	0.96	High frequency	Strong dependency Expected causal effect
Glucose → Glucose_Postpandrial	0.99	High frequency	Strong dependency Physiologically consistent
Steps → Heart_rate	0.97	High frequency	Strong dependency Expected causal effect
Carb_input → Glucose	0.67	Medium	Stable presence Metabolic relation

Source: Own elaboration, interpretation: high Frequency > 0.9, medium > 0.6.

**Table 4:** Comparative analysis of GRaSP Models.

Model	Number of Edges	Key Relationships	Implausible Edges	Interpretability and Clinical Consistency
*Model 1: Unconstrained GRaSP*	22	Hour → Date Hour → Glucose Hour → Glucose_postpandrial Calories → Heart_rate Calories → Steps Glucose → Glucose_postpandrial Glucose → Bolus_volume_delivered Heart_rate → Hour Heart_rate → Steps Total_time → Heart_rate Total_time → Date Total_time → Glucose Total_time—Basal_rate (No direction) Bolus_volume_delivered → Glucose_postpandrial Carb_input → Glucose Carb_input → Bolus_volume_delivered Carb_input—Basal_rate (No direction) Carb_input → Heart_rate Basal_rate → Hour Basal_rate → Heart_rate Basal_rate → Bolus_volume_delivered Basal_rate → Total_time	Yes	Low
*Model 2: Constrained GRaSP*	12	Hour → Glucose Hour → Steps Calories <- Steps Steps → Heart_rate Glucose → Glucose_postpandrial Glucose → Bolus_volume_delivered Bolus_volume_delivered → Glucose_postpandrial Carb_input → Glucose Carb_input → Bolus_volume_delivered Carb_input → Basal_rate Basal_rate → Bolus_volume_delivered Date → Glucose	Removed, but some persistent	High
*Model 3:Bootstrap GRaSP*	12 (stable subset)	Hour → Glucose Hour → Steps Steps → Calories Steps → Heart_rate Glucose → Glucose_postpandrial Glucose → Bolus_volume_delivered Bolus_volume_delivered → Glucose_postpandrial Carb_input → Glucose Carb_input → Bolus_volume_delivered Carb_input → Basal_rate Basal_rate → Bolus_volume_delivered Date → Glucose	Removed, but some persistent	High

## Data Availability

The dataset that was used in this project is the HUPA-UCM diabetes dataset, a public dataset published by J. Ignacio Hidalgo, Jorge Alvarado, Marta Botella, Aránzazu Aramendi, J. Manuel Velasco, and Óscar Garnica, and available at https://data.mendeley.com/datasets/3hbcscwz44/1, accessed on 27 February 2026 [29].

## Abbreviations

The following abbreviations are used in this manuscript:

AANN	Adversarial Artificial Neural Networks
AI	Artificial Intelligence
BIC	Bayesian Information Criterion
BMI	Body Mass Index
CC	Carbohydrate Counting
CPDAGs	Completed Partially Directed Acyclic Graphs
DAGs	Directed Acyclic Graphs
DRL	Deep Reinforcement Learning
FGES	Fast Greedy Equivalence Search
GFCI	Greedy Fast Causal Inference
GRaSP	Greedy Relaxations of the Sparsest Permutation
GSP	Generalized Sequential Pattern
HER	Electronic Health Records
IMSS	Instituto Mexicano del Seguro Social
MAG	Maximal Ancestral Graph
MLM	Machine Learning Models
PAG	Partial Ancestral Graphs
RL	Reinforcement Learning
SCM	Structural Causal Model
T1DM	Type 1 diabetes mellitus
TIR	Time in Range
XGBoost	eXtreme Gradient Boosting
